# Propensity-matched study of liposomal doxorubicin vs. doxorubicin in first-line DLBCL treatment: efficacy and safety

**DOI:** 10.3389/fmed.2026.1769270

**Published:** 2026-04-01

**Authors:** Lin Zeng, Chao Tang, Hao Cheng, Xianwei Peng, Xuanzhang Li, Xiaohong Tan, Jie Sun, Changxian Chen, Hong Cen, Chengcheng Liao

**Affiliations:** 1 Department of Hematology/Oncology, Guangxi Medical University Cancer Hospital, Nanning, China; 2 Department of Hematology, The Second Affiliated Hospital of Guangxi Medical University, Nanning, China; 3 State Key Laboratory of Targeting Oncology, Guangxi Medical University, Nanning, China

**Keywords:** diffuse large B-cell lymphoma, doxorubicin, overall survival, pegylated liposomal doxorubicin, progression-free survival

## Abstract

**Objective:**

Given the increasing use of pegylated liposomal doxorubicin (PLD) in diffuse large B-cell lymphoma (DLBCL) treatment due to its advantages in reducing adverse reactions, and the uncertainty surrounding its effective dose and corresponding efficacy in DLBCL, this study aims to compare it with the standard dose of conventional doxorubicin (DOX) in first-line DLBCL treatment.

**Methods:**

A retrospective propensity score-matched analysis of 512 DLBCL patients (2018–2023) compared PLD {stratified: low-dose [21.5 (5–25.5) mg/m^2^, *n* = 71] and high-dose [29.5 (25.5–40) mg/m^2^, *n* = 71]} with DOX {low-dose [32.4 (20–40) mg/m^2^, *n* = 47]} and standard-dose [49.0 (40–50) mg/m^2^, *n* = 323]. Endpoints included progression-free survival (PFS), overall survival (OS), and toxicity.

**Results:**

Overall PLD and DOX showed comparable 2-year PFS (74.3% vs. 69.6%, *P* = 0.479, Holm-Bonferroni *P* = 0.959) and OS (81.4% vs. 83.8%, *P* = 0.939, Holm-Bonferroni *P* = 0.959). High-dose PLD demonstrated significantly superior PFS vs. low-dose DOX (79.9% vs. 59.8%, *P* = 0.0066, Holm-Bonferroni *P* = 0.0132) and numerically higher PFS vs. overall DOX (81.0% vs. 70.5%, *P* = 0.354, Holm-Bonferroni *P* = 0.707), though this did not reach statistical significance. PLD significantly reduced hematologic toxicities (leukopenia: 7.7% vs. 56.7%, *P* < 0.001) and hepatic dysfunction (alanine aminotransferase elevation: 13.4% vs. 52.8%, *P* < 0.001), with similar cardiac/pneumonia events. Elderly patients (≥60 years) mirrored overall efficacy/safety trends.

**Conclusion:**

High-dose PLD [29.5 (25.5–40) mg/m^2^] offers a safer and potentially more effective alternative to standard DOX, while low-dose PLD maintains equivalent efficacy. These findings support optimized PLD dosing strategies in DLBCL therapy to enhance safety without compromising outcomes.

## Introduction

Diffuse large B-cell lymphoma (DLBCL) constitutes the most prevalent histological subtype of non-Hodgkin's lymphoma (NHL). It accounts for approximately 40% of all NHL cases worldwide (1). The R-CHOP therapeutic regimen (rituximab, cyclophosphamide, doxorubicin, vincristine, and prednisone) is universally recognized as the primary treatment modality for newly diagnosed DLBCL cases. Doxorubicin (DOX), as the cornerstone agent, has demonstrated well-established efficacy (2). However, its clinical utility is constrained by dose-limiting toxicities including cardiotoxicity, myelosuppression, and gastrointestinal reactions (3–5). Although 6–8 cycles of R-CHOP are conventionally recommended, treatment-related toxicities (particularly cardiotoxicity) may necessitate dose modifications or discontinuation, potentially compromising therapeutic outcomes (6, 7). To address these limitations, pegylated liposomal doxorubicin (PLD) has been introduced into clinical practice. PLD encapsulates DOX within polyethylene glycol-coated liposomal vesicles, thereby optimizing pharmacokinetic properties through prolonged plasma half-life, reduced cardiac tissue distribution, and enhanced tumor-selective passive targeting via the enhanced permeability and retention (EPR) effect. These characteristics mitigate cardiotoxicity risks while maintaining favorable efficacy and safety profiles in solid tumors such as breast and ovarian cancers (8, 9). This provides both theoretical rationale and clinical evidence for PLD application in DLBCL management.

Despite its therapeutic potential, significant evidence gaps persist regarding PLD's clinical application in DLBCL treatment: current evidence primarily derives from small-sample retrospective analyses or single-arm trials, lacking systematic comparisons with standard DOX regimens. Furthermore, the dose-response relationship of PLD in DLBCL remains undefined, particularly regarding long-term survival outcomes across different dosage ranges. While the standard DOX dose in R-CHOP is 40–50 mg/m^2^ (10), clinical practice often requires dose reduction to 20–40 mg/m^2^ due to toxicity (11). In contrast, PLD dosing strategies lack standardization, with optimal dosage ranges and long-term effects require further validation. Although NCCN guidelines recommend PLD for elderly DLBCL patients with cardiac comorbidities (12), its potential advantages in broader populations (e.g., younger patients) remain underexplored.

This study employs a retrospective cohort design utilizing real-world data with propensity score matching (PSM) methodology to systematically compare the efficacy and safety of PLD vs. DOX in first-line DLBCL treatment. PSM balances baseline characteristics to minimize confounding bias, thereby enhancing comparison validity. Specifically, PLD cohorts were stratified into low-dose (5–25.5 mg/m^2^) and high-dose (25.5–40 mg/m^2^) subgroups, while DOX groups were divided into low-dose (20–40 mg/m^2^) and standard-dose (40–50 mg/m^2^) subgroups. Long-term survival outcomes were analyzed across dosage groups, investigating whether PLD's prolonged half-life enables comparable/superior survival benefits at lower doses. Concurrently, we evaluated safety differentials between PLD and DOX, and explored their applicability across diverse populations. This provides evidence-based foundations for personalized DLBCL therapy.

This study analyzed clinical data from 512 DLBCL patients, employing propensity score matching (PSM) to balance baseline characteristics between PLD and DOX groups. The primary objectives were to elucidate the dose-survival relationship and evaluate their applicability across different patient subgroups. The findings may provide scientific evidence to guide personalized DLBCL treatment strategies, particularly in clinical decision-making requiring optimal efficacy-safety balance, ultimately facilitating more optimized therapeutic selection for patients.

## Materials and methods

### Study population and data collection

**Figure 1:**
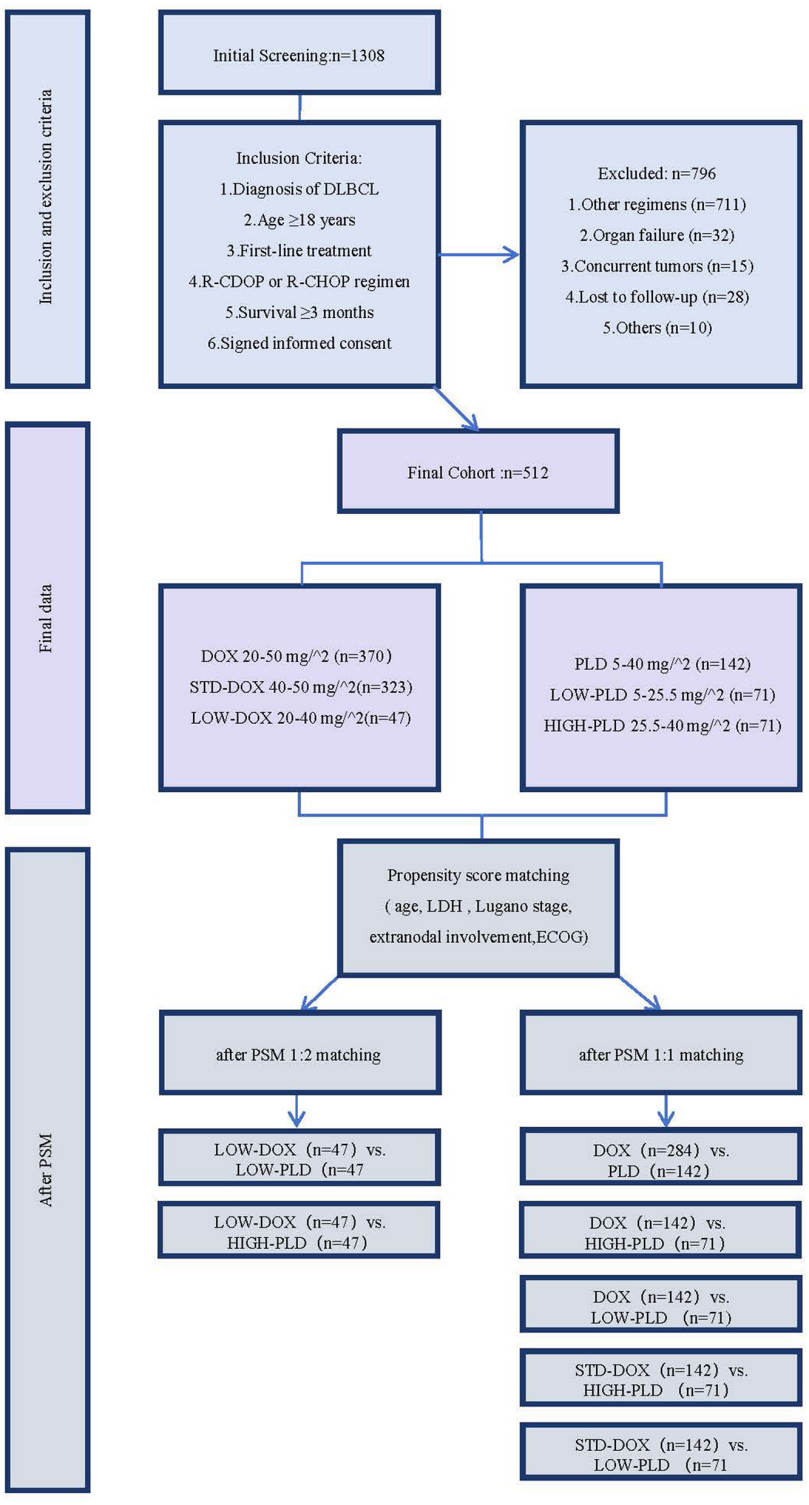
The general flowchart of this study. DLBCL, Diffuse Large B-cell Lymphoma; DOX, Doxorubicin group; PLD, Pegylated Liposomal Doxorubicin group; LOW-DOX, low-dose DOX subgroup; STD-DOX, standard-dose DOX subgroup; LOW-PLD, low-dose PLD subgroup; HIGH-PLD, high-dose PLD subgroup; PSM, Propensity Score Matching. DOX group: Patients treated with doxorubicin [46.2 (20–50) mg/m^2^]. Based on guideline-recommended standard doses (40–50 mg/m^2^ in R-CHOP), DOX was subdivided into: LOW-DOX: [32.4 (20–40) mg/m^2^]; STD-DOX: (40–50 mg/m^2^). PLD group: Patients treated with pegylated liposomal doxorubicin [25.5 (5–40) mg/m^2^]. Subgroups were stratified by median dose: LOW-PLD: [21.5 (5–25.5) mg/m^2^]; HIGH-PLD: [29.5 (25.5–40) mg/m^2^]. Propensity score matching covariates were selected based on the five key International Prognostic Index factors: age (≥60 years), Lugano stage (I–IV), LDH levels, number of extranodal involvement sites, and ECOG performance status.

We conducted a retrospective analysis of 512 DLBCL patients treated at Guangxi Medical University Cancer Hospital between January 1, 2018 and December 31, 2023 (Figure 1). Data collection encompassed: (1) demographic characteristics (age, sex); (2) disease features (diagnosis date, tumor location, histological classification, number of nodal/extranodal involvement sites, bone marrow infiltration); (3) laboratory parameters (serum lactate dehydrogenase, LDH); and (4) treatment-related information (first-line regimen, treatment response, performance status scores). Trained registrars extracted all data from medical records using WHO International Classification of Diseases for Oncology (ICD-O) codes (9680, 9684) for standardization. Inclusion criteria were:(1) Histopathological and immunohistochemical confirmation of DLBCL; (2) Age ≥18 years; (3) Indication for first-line therapy; (4) Treatment with either R-CDOP (rituximab, cyclophosphamide, liposomal doxorubicin, vincristine, prednisone) or R-CHOP (rituximab, cyclophosphamide, doxorubicin hydrochloride, vincristine, prednisone); (5) Completed at least one cycle of planned chemotherapy; (6) Signed informed consent from patients or legal representatives. Exclusion criteria comprised: (1) Alternative chemotherapy regimens (CHOP/R-CEOP, R-miniCHOP, R-DA-EPOCH, etc.); (2) Severe cardiac/hepatic/renal dysfunction (NYHA class ≥III, Child-Pugh grade ≥B, or eGFR < 30 mL/min/1.73 m^2^); (3) History of/concurrent malignancies (except non-melanoma skin cancer); (4) Pregnancy or lactation; (5) Psychiatric/cognitive disorders precluding treatment compliance. Survival follow-up continued through February 1, 2026, with surviving patients censored in survival analyses. Some treatment cycles utilized PLD while others used DOX in combination regimens. All participants or their legal representatives provided written informed consent for participation in the study and the use of their anonymized data for research purposes. The study protocol was approved by the Institutional Review Board of Guangxi Medical University Cancer Hospital (Approval No. KY2025039), with all data anonymized to protect patient confidentiality.

### Treatment regimens

DOX group: Patients received standard R-CHOP regimen: Rituximab 375 mg/m^2^ IV infusion (day 1), Cyclophosphamide 650–750 mg/m^2^ IV (day 1), Doxorubicin hydrochloride 20–50 mg/m^2^ IV (day 1), Vincristine 1.4 mg/m^2^ (capped at 2 mg) IV (day 1), Prednisone 60–100 mg PO (days 1–5). Cycles repeated every 21 days.

DOX dosing: 20–50 mg/m^2^ (mean ± SD: 46.2 ± 6.0 mg/m^2^, range: 20.8–50.0 mg/m^2^, *n* = 370). Per established R-CHOP protocols, the standard doxorubicin dose is 50 mg/m^2^. However, doses of 40–50 mg/m^2^ are commonly administered in clinical practice and have been used as the standard comparator in previous studies (13, 14). In this study, we defined standard-dose DOX (STD-DOX) as 40–50 mg/m^2^ to reflect real-world practice patterns, while doses < 40 mg/m^2^ were classified as low-dose (LOW-DOX). DOX subgroups were: LOW-DOX: 20–40 mg/m^2^ (32.4 ± 5.7 mg/m^2^, range: 20.8–39.7 mg/m^2^, *n* = 47); STD-DOX: 40–50 mg/m^2^ (49.0 ± 2.4 mg/m^2^, range: 40.2–50.0 mg/m^2^, *n* = 323). The LOW-DOX subgroup comprised patients who initiated standard R-CHOP but received individualized doxorubicin dose reductions based on clinical judgment (e.g., advanced age, borderline performance status, or physician preference), rather than patients enrolled in pre-specified dose-attenuated protocols such as R-miniCHOP. This distinction is clinically relevant, as individualized dose adjustments within R-CHOP represent common real-world practice patterns that differ from protocol-mandated attenuated regimens.

PLD group: Patients received R-CDOP regimen: Rituximab 375 mg/m^2^ IV infusion (day 1), Cyclophosphamide 650–750 mg/m^2^ IV (day 1), Pegylated liposomal doxorubicin 5–40 mg/m^2^ IV (day 1), Vincristine 1.4 mg/m^2^ (capped at 2 mg) IV (day 1), Prednisone 60–100 mg PO (days 1–5). Cycles repeated every 21 days.

PLD dosing: 5–40 mg/m^2^ (mean ± SD: 25.5 ± 6.0 mg/m^2^, range: 5.6–40.0 mg/m^2^, *n* = 142). Using median dose stratification, PLD subgroups were: LOW-PLD: 5.0–25.5 mg/m^2^ (21.5 ± 5.1 mg/m^2^, range: 5.6–25.4 mg/m^2^, *n* = 71); HIGH-PLD: 25.5–40.0 mg/m^2^ (29.5 ± 3.8 mg/m^2^, range: 25.5–40.0 mg/m^2^, *n* = 71).

### Response assessment criteria

Treatment response was evaluated according to the 2014 Lugano classification for lymphoma response criteria (15, 16). Primary endpoints included: Progression-free survival (PFS): Time from diagnosis to disease progression, refractory disease, or death from any cause, whichever occurred first; Overall survival (OS): Time from diagnosis to death from any cause. Evaluations were performed after cycles 2, 4, 6, and at treatment completion, including: Clinical history and physical examination, Whole-body CT scans (chest/abdomen/pelvis), Digital whole-body color Doppler ultrasound, PET-CT imaging, Bone marrow aspiration/biopsy. Adverse events were graded per CTCAE version 5.0. Cardiotoxicity is defined as any of the following: (1) symptomatic heart failure (NYHA class II–IV); (2) an asymptomatic decline in left ventricular ejection fraction (LVEF) of more than 10 percentage points from baseline, with a final LVEF below 50%; (3) clinically significant arrhythmias requiring therapeutic intervention; and (4) acute coronary syndrome temporally associated with chemotherapy administration. Baseline LVEF was assessed by transthoracic echocardiography (TTE) within 30 days prior to the initiation of chemotherapy. LVEF data were available for 350 of 512 patients (68.4%). The distribution of baseline LVEF was similar across treatment groups (mean 68.1% in the DOX group vs. 67.3% in the PLD group, SMD = 0.17). Pneumonia is defined as the presence of new pulmonary infiltrates on chest imaging (CT or X-ray), accompanied by clinical symptoms (temperature >38.0 °C, cough, dyspnea, or purulent sputum) and/or positive microbiological culture results. The diagnosis is based on the attending physician's clinical assessment as documented in the medical records.

### Statistical analysis

Data processing and analysis were performed using R software (version 4.2.1) and SPSS (version 22.0). To minimize confounding bias and simulate randomized controlled trial (RCT) conditions, propensity score matching (PSM) was implemented using the R package “MatchIt” (17). The optimal matching algorithm (18) was employed with a caliper width of 0.05, using 1:1 or 1:2 matching ratios (each PLD-treated patient matched with 1–2 DOX-treated patients having similar propensity scores). A pairwise PSM strategy, rather than a simultaneous four-group matching approach, was adopted for the following three reasons. First, the dose subgroups were subject to confounding by indication: patients who received reduced doses (LOW-DOX) were systematically older (53.2% vs. 27.9% aged ≥60 years, SMD = 0.53) and had worse performance status (38.3% vs. 20.4% with ECOG score ≥2, SMD = 0.40) than those who received standard-dose treatment, as dose reductions were clinically determined by patient frailty or comorbidities rather than being randomly assigned. These inherent structural differences severely restrict the common support region required for the valid estimation of four-group propensity scores. Second, the smallest subgroup (LOW-DOX, *n* = 47) had an insufficient sample size to support the stable estimation of multinomial propensity scores. Third, the core clinical questions addressed by this study are inherently pairwise in nature. To correct for multiple testing, the Holm-Bonferroni method was applied to all six pairwise comparisons. The covariates included in the matching were key factors of the International Prognostic Index (IPI): age (≥60 years), Lugano stage (I–IV), lactate dehydrogenase (LDH) level, number of extranodal involvement sites, and Eastern Cooperative Oncology Group (ECOG) performance status score. The post-matching sample sizes and standardized mean differences (SMD) are reported in the Results section. Person-years of follow-up were calculated as the sum of individual observation time, which was defined as the period from the initiation of treatment to the date of event (disease progression, disease relapse, or all-cause death) or the date of the last follow-up (censoring), whichever came first. Chi-square tests were used to compare the balance of baseline variables and the incidence rates of adverse events between groups before and after matching. The adequacy of matching was confirmed when between-group differences for all covariates yielded a *P-value* > 0.05. The Kaplan-Meier method with log-rank test was used for survival analyses, to compare PFS and OS between groups. Missing data were processed using the multiple imputation method. A two-sided significance level of α = 0.05 was set for all statistical tests. The primary comparisons of this study were HIGH-PLD vs. STD-DOX, and LOW-PLD vs. STD-DOX, as these directly answer the core clinical question of whether PLD-based R-CDOP regimen achieves comparable efficacy to the standard DOX-based R-CHOP regimen. The remaining four pairwise comparisons (HIGH-PLD vs. LOW-DOX, LOW-PLD vs. LOW-DOX, HIGH-PLD vs. LOW-PLD, and STD-DOX vs. LOW-DOX) were defined as exploratory analyses. For the primary comparisons, a two-sided *P-value* < 0.05 was considered statistically significant. Holm-Bonferroni-adjusted *P-values* were also reported for all six comparisons, to comprehensively evaluate the robustness of the study findings. To address the potential confounding effect of baseline cardiac function, we performed a sensitivity analysis restricted to the 350 patients with available pre-treatment left ventricular ejection fraction (LVEF) data. In this sensitivity analysis, LVEF was added as an additional matching covariate, along with age, LDH level, disease stage, extranodal involvement, and ECOG performance status. We first compared the baseline characteristics between patients with and without available LVEF data, to verify whether the missing data pattern conformed to the missing-at-random (MAR) assumption. In addition, we constructed multivariable Cox proportional hazards models adjusted for LVEF and all baseline covariates, to estimate the adjusted hazard ratios.

## Results

### Patient characteristics

This study enrolled 512 DLBCL patients treated between January 1, 2018 and December 31, 2023. The age range was 18–82 years (median 56 years), including 282 males (55.1%) and 230 females (44.9%). 74.2% had ECOG performance status ≤ 1, and 56.4% presented with Lugano stage III–IV disease. Median follow-up was 29 months (range: 2–98). Treatment groups by regimen and dose: DOX group (*n* = 370): STD-DOX [49.0 (40–50) mg/m^2^, *n* = 323] and LOW-DOX [32.4 (20–40) mg/m^2^, *n* = 47]; PLD group (*n* = 142): HIGH-PLD [29.5 (25.5–40) mg/m^2^, *n* = 71] and LOW-PLD [21.5 (5–25.5) mg/m^2^, *n* = 71].

PSM used 1:2 matching (1:1 for LOW-DOX due to limited samples). Final matched pairs: DOX (*n* = 284) vs. PLD (*n* = 142) (Table 1A); DOX (*n* = 142) vs. HIGH-PLD (*n* = 71) (Supplementary Table S1) and LOW-PLD (*n* = 71) (Supplementary Table S2); STD-DOX (*n* = 142) vs. HIGH-PLD (*n* = 71) (Supplementary Table S3) and LOW-PLD (*n* = 71) (Supplementary Table S4); LOW-DOX (*n* = 47) vs. HIGH-PLD (*n* = 47) (Supplementary Table S5) and LOW-PLD (*n* = 47) (Supplementary Table S6). Pre-PSM groups showed no significant differences in gender, cell origin or Lugano stage (*P* > 0.05). Baseline left ventricular ejection fraction (LVEF) availability and levels were well-balanced across all dose subgroups (Table 1B). The proportions of patients with available LVEF data were 63.4%, 59.2%, 70.9%, and 72.3% in the HIGH-PLD, LOW-PLD, STD-DOX, and LOW-DOX subgroups, respectively. The mean ± SD of baseline LVEF in each subgroup was 67.4 ± 3.6%, 67.1 ± 3.4%, 68.2 ± 3.3%, and 67.8 ± 3.5%, with an overall standardized mean difference (SMD) of 0.17 (Table 1B). Significant intergroup differences (*P* < 0.05) existed for: Age (≥60 years) and ECOG between DOX vs. PLD (Table 1). Age (≥60 years) and ECOG between DOX vs. LOW-PLD (Supplementary Table S2). Age (≥60 years), Number of extranodal sites and ECOG between STD-DOX vs. LOW-PLD (Supplementary Table S4). LDH levels between LOW-DOX vs. HIGH-PLD (Supplementary Table S5). After PSM adjustment for covariates (gender, age, LDH, Lugano stage, Number of extranodal sites, ECOG), baseline characteristics were balanced across groups (*P* > 0.05).

**Table 1A:** Baseline data of the DOX group and the PLD group before and after PSM 1:2 matching, *n* (%).

Baseline Characteristics	Original queue						1:2 matching queue					
	DOX	%	PLD	%	*P*	SMD	DOX	%	PLD	%	*P*	SMD
*N*	370		142				284		142			
Male sex	206	55.7	76	53.5	0.734	0.043	157	55.3	76	53.5	0.81	0.035
>60 years												
No	255	68.9	78	54.9	0.004	0.291	177	62.3	78	54.9	0.173	0.151
Yes	115	31.1	64	45.1			107	37.7	64	45.1		
Gene expression profiling												
GCB	207	55.9	72	50.7	0.381	0.103	160	56.3	72	50.7	0.472	0.091
non-GCB	121	32.7	52	36.6			96	33.8	52	36.6		
Unknown	42	11.4	18	12.7			28	9.9	18	12.7		
Lactate dehydrogenase												
Normal	192	51.9	83	58.5	0.217	0.132	162	57.0	83	58.5	0.862	0.029
Elevated	178	48.1	59	41.5			122	43.0	59	41.5		
Lugano stage												
I-II	165	44.6	58	40.8	0.505	0.076	115	40.5	58	40.8	1	0.007
III-IV	205	55.4	84	59.2			169	59.5	84	59.2		
Number of extranodal sites												
0–1	293	79.2	101	71.1	0.068	0.187	222	78.2	101	71.1	0.139	0.162
>2	77	20.8	41	28.9			62	21.8	41	28.9		
ECOG												
0–1	286	77.3	94	66.2	0.014	0.248	200	70.4	94	66.2	0.437	0.091
2-−5	84	22.7	48	33.8			84	29.6	48	33.8		

DOX, Doxorubicin group; PLD, Pegylated Liposomal Doxorubicin group; SMD, Standardized Mean Difference; ECOG, Eastern Cooperative Oncology Group; GCB, germinal center B-cell. Original queue: Pre-matching baseline characteristics. 1:2 matched queue: Post-matching characteristics after adjusting for covariates [age (>60 years), LDH, Lugano stage, Number of extranodal sites, ECOG).

Pre-matching significant differences in age (>60 years) and ECOG (*P* < 0.05); post-matching balance achieved (*P* > 0.05). Post-matching SMD < 0.1 for sex, LDH, Lugano stage, ECOG; SMD < 0.2 for age (>60 years) and Number of extranodal site.

**Table 1B:** Baseline left ventricular ejection fraction (LVEF) availability and mean levels in different dose subgroups, *n* (%).

Variable	HIGH-PLD	LOW-PLD	STD-DOX	LOW-DOX	SMD
LVEF available, *n* (%)	45 (63.4%)	42 (59.2%)	229 (70.9%)	34 (72.3%)	–
LVEF, mean ± SD (%)	67.4 ± 3.6	67.1 ± 3.4	68.2 ± 3.3	67.8 ± 3.5	0.17

LVEF, left ventricular ejection fraction; SD, standard deviation.

The table shows the number and percentage of patients with available baseline LVEF data, as well as the mean ± SD of baseline LVEF levels in each dose subgroup; the overall SMD of baseline LVEF levels across all subgroups was 0.17, suggesting a balanced distribution of baseline cardiac function among the four dose subgroups.

### Survival outcome comparisons

**Figure 2:**
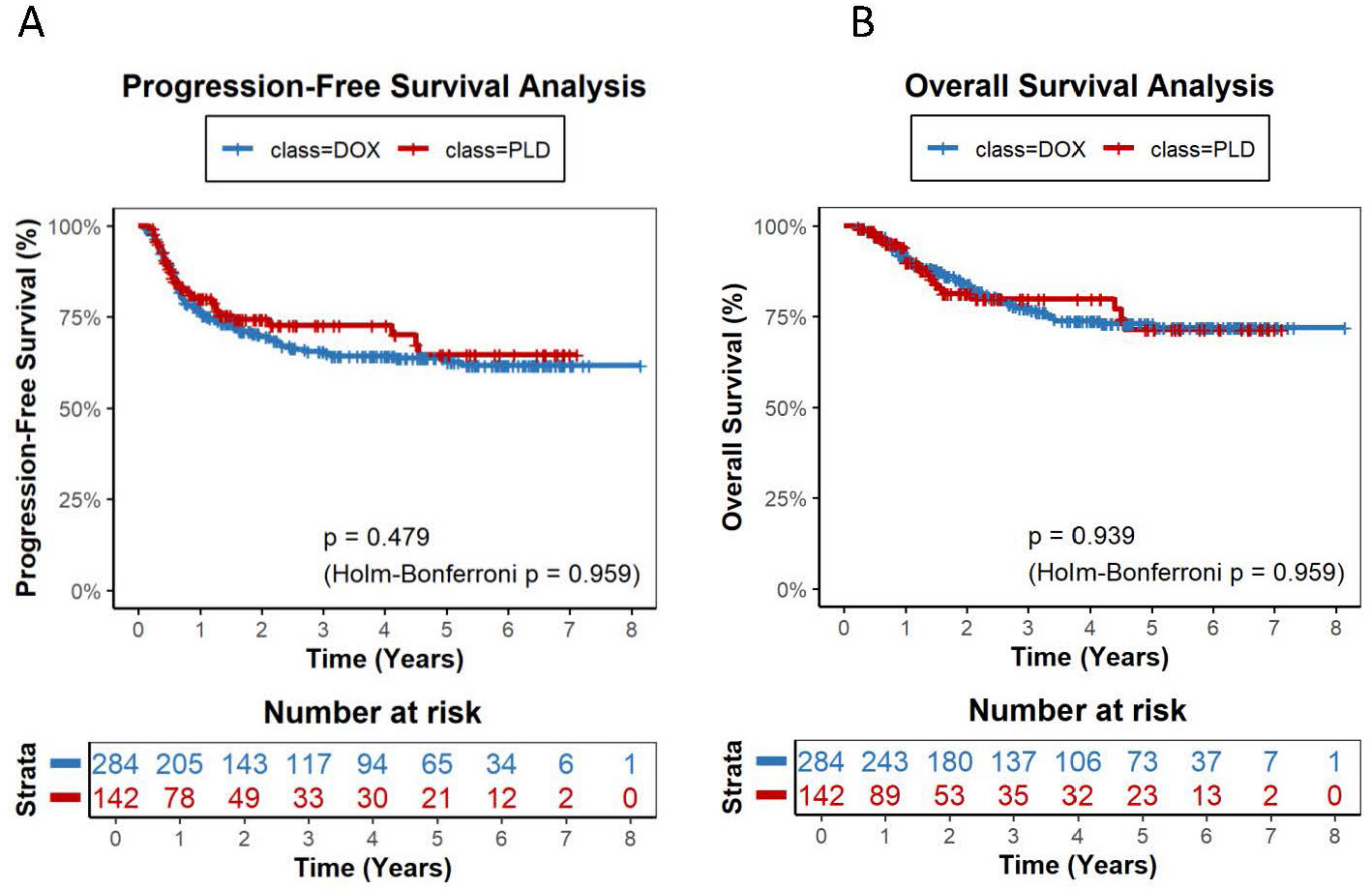
Kaplan-Meier survival curves comparing PLD vs. DOX (1:2 matched) for progression-free survival (PFS) and overall survival (OS). **(A)** Two-year PFS rates: 74.3% (PLD) vs. 69.6% (DOX), *P* = 0.479, Holm-Bonferroni *P* = 0.959. **(B)** Two-year OS rates: 81.4% (PLD) vs. 83.8% (DOX), *P* = 0.939, Holm-Bonferroni *P* = 0.959.

**Figure 3:**
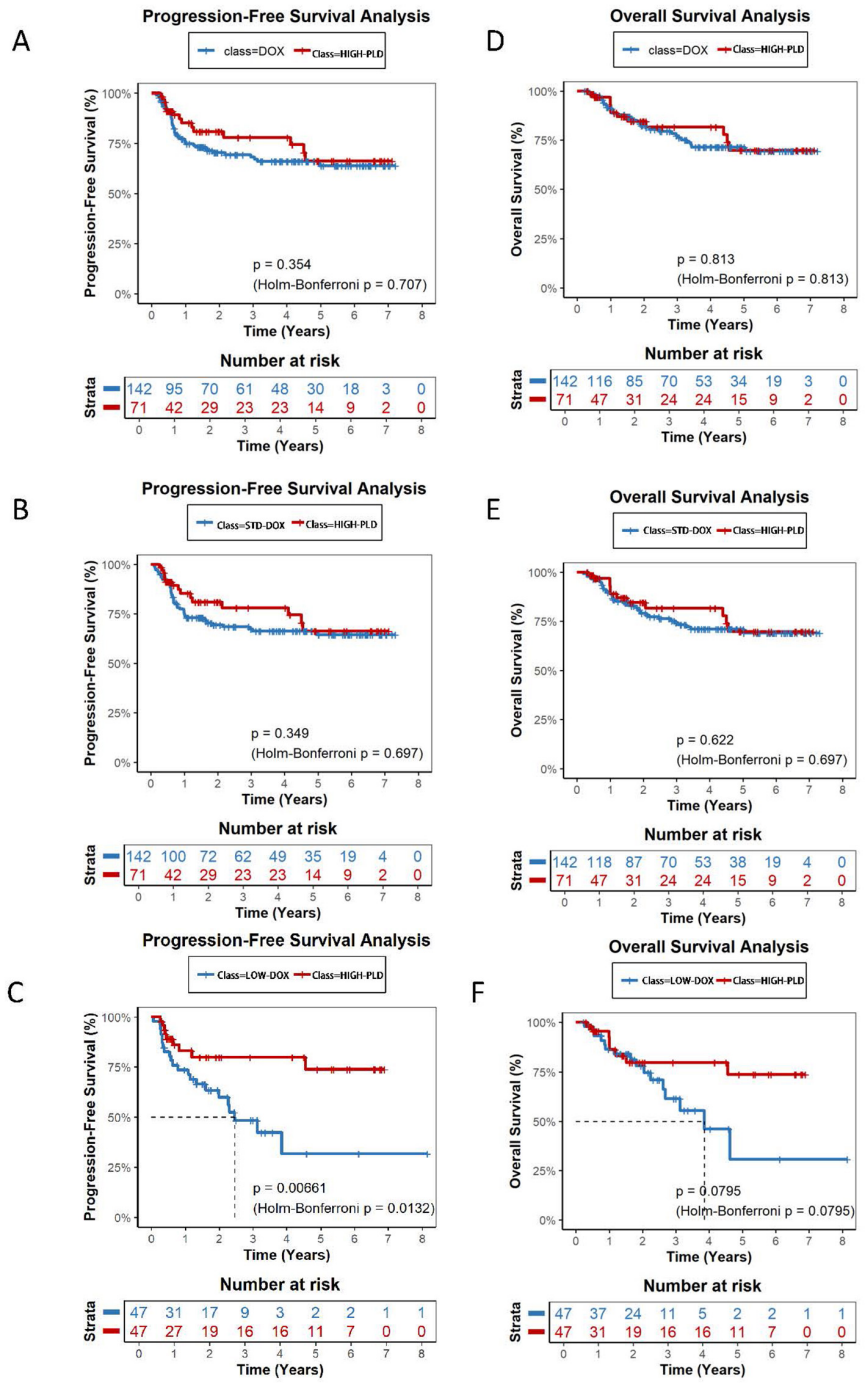
Kaplan-Meier survival curves comparing HIGH-PLD vs.: DOX (1:2 matched) for PFS(A) and OS(D); STD-DOX (1:2 matched) for PFS(B) and OS(E); LOW-DOX (1:1 matched) for PFS(C) and OS(F). **(A)** HIGH-PLD vs. DOX: 2-year PFS 81.0% vs. 70.5%, *P* = 0.354, Holm-Bonferroni *P* = 0.707. **(B)** HIGH-PLD vs. STD-DOX: 2-year PFS 81.0% vs. 69.4%, *P* = 0.349, Holm-Bonferroni *P* = 0.697. **(C)** HIGH-PLD vs. LOW-DOX: 2-year PFS 79.9% vs. 59.8%, *P* = 0.0066, Holm-Bonferroni *P* = 0.0132. **(D)** HIGH-PLD vs. DOX: 2-year OS 81.4% vs. 83.8%, *P* = 0.813, Holm-Bonferroni *P* = 0.813. **(E)** HIGH-PLD vs. STD-DOX: 2-year OS 84.6% vs. 82.4%, *P* = 0.622, Holm-Bonferroni *P* = 0.697. **(F)** HIGH-PLD vs. LOW-DOX: 2-year OS 84.6% vs. 79.2%, *P* = 0.0795, Holm-Bonferroni *P* = 0.0795.

**Figure 4:**
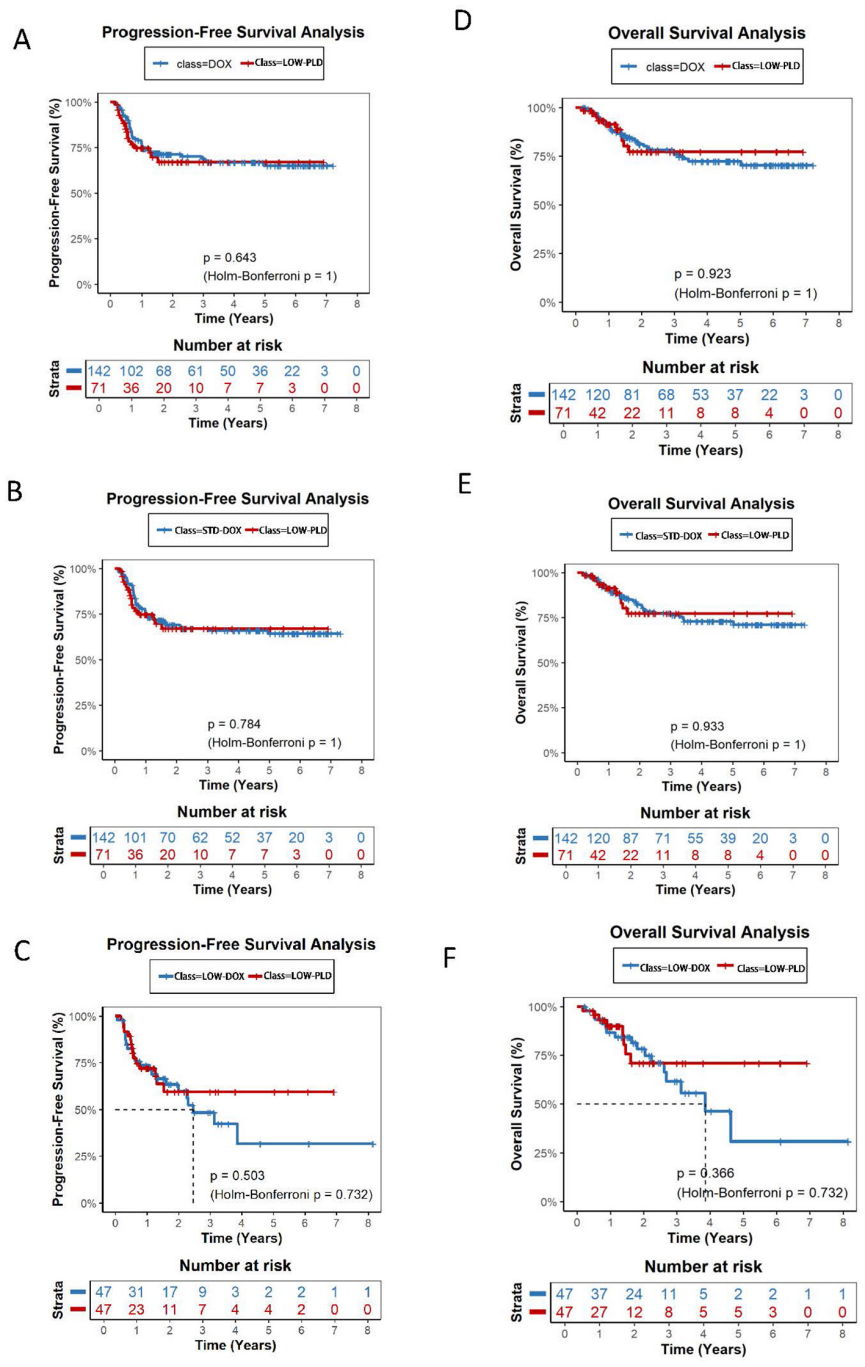
Kaplan-Meier survival curves comparing LOW-PLD vs.: DOX (1:2 matched) for PFS(A) and OS(D); STD-DOX (1:2 matched) for PFS(B) and OS(E); LOW-DOX (1:1 matched) for PFS(C) and OS(F). **(A)** LOW-PLD vs. DOX: 2-year PFS 67.1% vs. 71.2%, *P* = 0.643, Holm-Bonferroni *P* = 1. **(B)** LOW-PLD vs. STD-DOX: 2-year PFS 67.1% vs. 68.1%, *P* = 0.784, Holm-Bonferroni *P* = 1. **(C)** LOW-PLD vs. LOW-DOX: 2-year PFS 59.5%% vs. 59.8%, *P* = 0.503, Holm-Bonferroni *P* = 0.732. **(D)** LOW-PLD vs. DOX: 2-year OS 77.2% vs. 81.2%, *P* = 0.923, Holm-Bonferroni *P* = 1. **(E)** LOW-PLD vs. STD-DOX: 2-year OS 77.2% vs. 81.3%, *P* = 0.933, Holm-Bonferroni *P* = 1. **(F)** LOW-PLD vs. LOW-DOX: 2-year OS 70.9% vs. 78.2%, *P* = 0.366, Holm-Bonferroni *P* = 0.732.

Over a total follow-up of 1,519.2 person-years for OS and 1,341.2 person-years for PFS, 101 deaths and 151 progression or relapse events were observed across the entire cohort. The median follow-up for OS was 3.18 years in the STD-DOX group, 1.61 years in HIGH-PLD, 1.24 years in LOW-PLD, and 2.02 years in LOW-DOX (Table 2). Kaplan-Meier analysis revealed no significant differences in overall PFS or OS between PLD and DOX groups with 2-year PFS rates of 74.3% vs. 69.6% (*P* = 0.479, Holm-Bonferroni *P* = 0.959) and 2-year OS rates of 81.4% vs. 83.8% (*P* = 0.939, Holm-Bonferroni *P* = 0.959) (Figure 2). HIGH-PLD demonstrated comparable PFS vs. DOX group and STD-DOX subgroup: 2-year PFS: 81.0% vs. 70.5% (*P* = 0.354, Holm-Bonferroni *P* = 0.707) vs. DOX, 81.0% vs. 69.4% (*P* = 0.349, Holm-Bonferroni *P* = 0.697) vs. STD-DOX, but demonstrated significantly superior PFS vs. LOW-DOX subgroup: 79.9% vs. 59.8% (*P* = 0.0066, Holm-Bonferroni *P* = 0.0132) vs. LOW-DOX (Figures 3A–C). For OS, HIGH-PLD showed comparable trends vs. all DOX subgroups: 2-year OS: 81.4% vs. 83.8% (*P* = 0.813, Holm-Bonferroni *P* = 0.813) vs. DOX, 84.6% vs. 82.4% (*P* = 0.622, Holm-Bonferroni *P* = 0.697) vs. STD-DOX, 84.6% vs. 79.2% (*P* = 0.0795, Holm-Bonferroni *P* = 0.0795) vs. LOW-DOX, though differences were non-significant (Figures 3D–F). LOW-PLD showed comparable PFS/OS to all DOX subgroups: 2-year PFS: 67.1% vs. 71.2% (*P* = 0.643, Holm-Bonferroni *P* = 1) vs. DOX, 67.1% vs. 68.1% (*P* = 0.784, Holm-Bonferroni *P* = 1) vs. STD-DOX, 59.5% vs. 59.8% (*P* = 0.503, Holm-Bonferroni *P* = 0.732) vs. LOW-DOX (Figures 4A–C). Two-year OS: 77.2% vs. 81.2% (*P* = 0.923, Holm-Bonferroni *P* = 1) vs. DOX, 77.2% vs. 81.3% (*P* = 0.933, Holm-Bonferroni *P* = 1) vs. STD-DOX, 70.9% vs. 78.2% (*P* = 0.366, Holm-Bonferroni *P* = 0.732) vs. LOW-DOX (Figures 4D–F). Among the 47 patients in the LOW-DOX subgroup (mean dose 32.4 ± 5.8 mg/m^2^, range 20.8–39.7), the clinical profile was consistent with a frailer patient population: 36.2% were aged ≥70 years (vs. 6.5% in STD-DOX), 38.3% had ECOG performance status ≥2, 27.3% had baseline anemia (hemoglobin < 100 g/L), and 34.1% had baseline neutropenia (ANC < 1.5 × 10^9^/L). Notably, baseline LVEF was normal in all evaluable patients (mean 67.4 ± 3.5%, all ≥60%), indicating that cardiac dysfunction was not a primary indication for dose reduction (Supplementary Table S7). Sensitivity analysis restricted to 350 patients with available baseline LVEF data yielded consistent results (Supplementary Table S8). After including LVEF as an additional matching covariate, the direction and magnitude of all pairwise comparisons remained unchanged. Five of six PFS comparisons and five of six OS comparisons showed identical statistical conclusions to the primary analysis (all adjusted *P* > 0.05 after Holm-Bonferroni correction). Multivariable Cox regression adjusting for LVEF and all baseline covariates confirmed that LVEF did not materially alter the estimated hazard ratios (Supplementary Table S7). These findings support the robustness of the primary analysis despite the incomplete availability of LVEF data.

**Table 2:** Progression-free survival and overall survival events, person-years of follow-up, event rates, and median follow-up duration across different dose subgroups.

Subgroup	*N*	PFS Events	PFS Person-Years	OS Events	OS Person-Years	PFS Event Rate^*^	OS Event Rate^*^	Median FU (OS), yr
HIGH-PLD	71	14	176.9	12	186.9	7.9	6.4	1.61
LOW-PLD	71	19	114.6	10	127.5	16.6	7.8	1.24
STD-DOX	323	96	962.3	63	1,099.2	10.0	5.7	3.18
LOW-DOX	47	22	87.4	16	105.6	25.2	15.2	2.02
Total	512	151	1,341.2	101	1,519.2	11.3	6.6	2.56

PFS, progression-free survival; OS, overall survival; HIGH-PLD, high-dose pegylated liposomal doxorubicin; LOW-PLD, low-dose pegylated liposomal doxorubicin; STD-DOX, standard-dose doxorubicin; LOW-DOX, low-dose doxorubicin; FU, follow-up.

Event rates are presented as the number of events per 100 person-years of follow-up. The symbol

^*^ denotes “Event rate per 100 person-years”.

### Subgroup analysis

**Figure 5:**
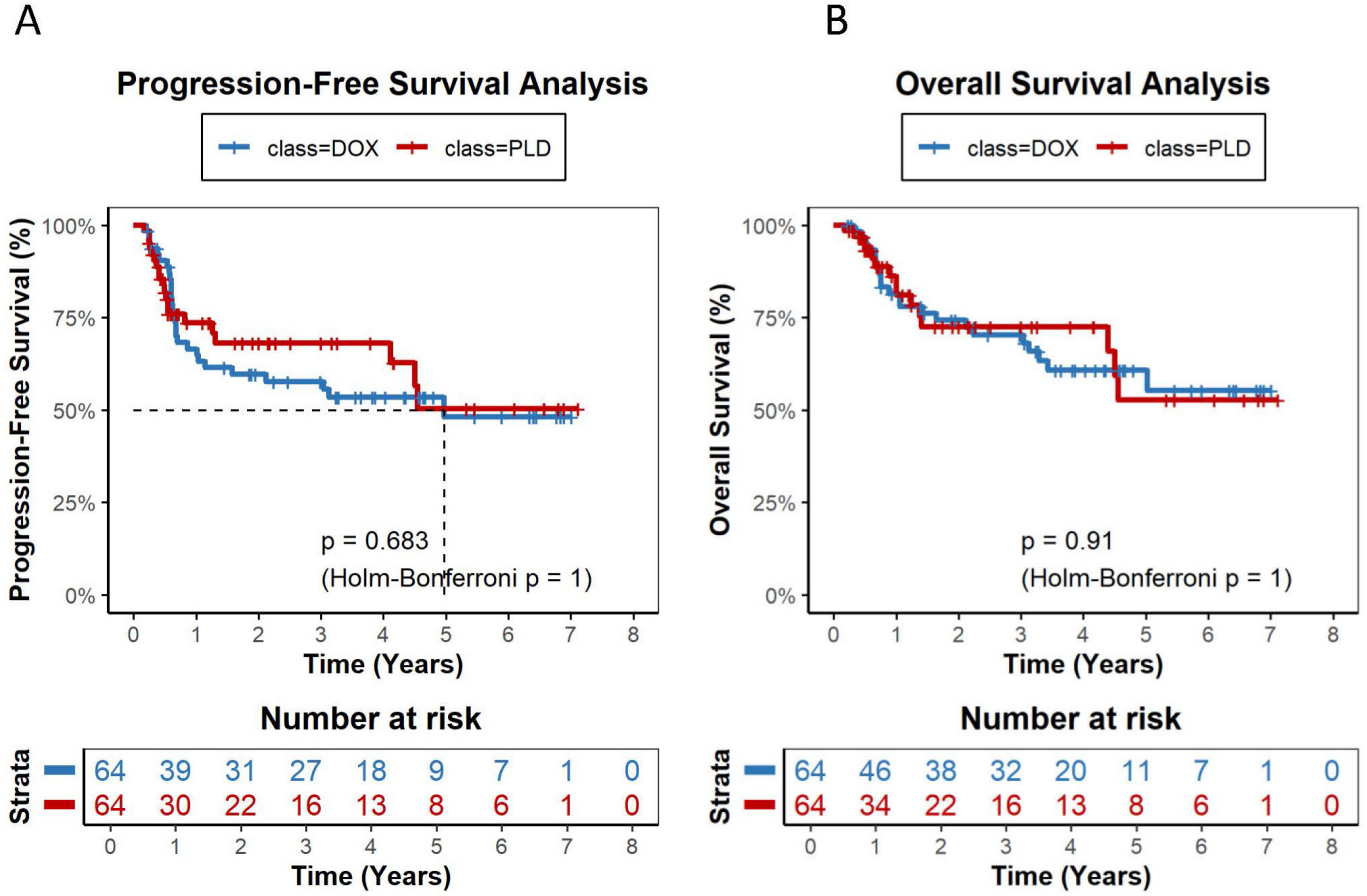
Subgroup analysis in elderly patients (≥60 years): Kaplan-Meier curves comparing PLD vs. DOX (1:1 matched) for PFS(A) and OS(B). **(A)** 2-year PFS: 68.1% (PLD) vs. 57.8% (DOX), *P* = 0.683, Holm-Bonferroni *P* = 1. **(B)** 2-year OS: 72.6% (PLD) vs. 72.4% (DOX), *P* = 0.91, Holm-Bonferroni *P* = 1.

To further evaluate whether PLD-based R-CDOP regimen (replacing conventional doxorubicin) provides survival benefits for elderly patients (≥60 years), we conducted a subgroup analysis. Among 179 elderly patients (≥60 years), 1:1 propensity score matching (PSM) yielded 64 matched pairs (DOX n = 64 vs. PLD *n* = 64). Kaplan-Meier analysis demonstrated 2-year PFS rates of 68.1% (PLD) vs. 57.8% (DOX) (*P* = 0.683, Holm-Bonferroni *P* = 1); and 2-year OS rates of 72.6% vs. 72.4% (*P* = 0.91, Holm-Bonferroni *P* = 1) (Figure 5). Although PLD showed numerically superior PFS and OS vs. DOX, the intergroup differences were not statistically significant.

### Safety analysis

Post-PSM, the DOX group showed significantly higher incidences of hematologic toxicities (leukopenia, neutropenia, anemia, thrombocytopenia) and hepatic dysfunction (ALT/AST elevation) vs. PLD (all *P* < 0.05). In the matched cohorts (DOX *n* = 284 vs. PLD *n* = 142): Leukopenia: 56.7% vs. 7.7% (*P* < 0.001), Neutropenia: 56.7% vs. 7.0% (*P* < 0.001), Anemia: 52.1% vs. 9.9% (*P* < 0.001), Thrombocytopenia: 11.3% vs. 2.8% (*P* = 0.006), ALT elevation: 52.8% vs. 13.4% (*P* < 0.001), AST elevation: 49.3% vs. 2.8% (*P* < 0.001). For other adverse events: Pneumonia incidence was numerically higher in the PLD group (21.8% vs. 19.4%, *P* = 0.639), Cardiac toxicity, defined as any of the following: symptomatic heart failure, asymptomatic LVEF decline ≥10% from baseline to < 50%, clinically significant arrhythmias, or myocardial ischemia, was observed in 24.6% of DOX patients vs. 17.6% of PLD patients (*P* = 0.128). The majority of cardiac events were Grade 1–2 (DOX: 18.3%; PLD: 14.1%), with Grade 3–4 events occurring in 6.3% (DOX) and 3.5% (PLD) of patients, respectively, though neither difference reached statistical significance (Table 3).

**Table 3:** Comparison of adverse events between matched DOX (*n* = 284) and PLD (*n* = 142) groups.

Adverse events	Original queue				1:2 matching queue			
	DOX	PLD	*P*	SMD	DOX	PLD	*P*	SMD
Pneumonia, *n* (%)	71 (19.2)	31 (21.8)	0.585	0.065	65 (19.4)	31 (21.8)	0.639	0.061
Leukopenia, *n* (%)	213 (57.6)	11 (7.7)	< 0.001	1.254	161 (56.7)	11 (7.7)	< 0.001	1.229
Neutropenia, *n* (%)	209 (56.5)	10 (7.0)	< 0.001	1.253	161 (56.7)	10 (7.0)	< 0.001	1.259
Anemia, n (%)	188 (50.8)	14 (9.9)	< 0.001	0.995	148 (52.1)	14 (9.9)	< 0.001	1.027
Thrombocytopenia, *n* (%)	47 (12.7)	4 (2.8)	0.001	0.376	32 (11.3)	4 (2.8)	0.006	0.335
ALT elevation, *n* (%)	210(56.8)	19(13.4)	< 0.001	1.021	150(52.8)	19(13.4)	< 0.001	0.923
AST elevation, *n* (%)	200 (54.1)	4 (2.8)	< 0.001	1.38	140 (49.3)	4 (2.8)	< 0.001	1.276
Cardiac toxicity, *n* (%)	85 (23.0)	25 (17.6)	0.229	0.134	70 (24.6)	25 (17.6)	0.128	0.173

DOX, Doxorubicin group; PLD, Pegylated Liposomal Doxorubicin group; SMD, Standardized Mean Difference; ALT, alanine aminotransferase; AST, aspartate aminotransferase.

Significant differences (*P* < 0.05): leukopenia: 56.7% (DOX) vs. 7.7% (PLD), *P* < 0.001; Neutropenia: 56.7% (DOX) vs. 7.0% (PLD), *P* < 0.001; Anemia: 52.1% (DOX) vs. 9.9% (PLD), *P* < 0.001; Thrombocytopenia: 11.3% (DOX) vs. 2.8% (PLD), *P* = 0.006; ALT elevation: 52.8% (DOX) vs. 13.4% (PLD), *P* < 0.001; AST elevation: 49.3% (DOX) vs. 2.8% (PLD), *P* < 0.001. Non-significant trends: Pneumonia: 19.4% (DOX) vs. 21.8% (PLD), *P* = 0.639; Cardiac toxicity: 24.6% (DOX) vs. 17.6% (PLD), *P* = 0.128.

## Discussion

Diffuse large B-cell lymphoma (DLBCL), the most common aggressive non-Hodgkin lymphoma in adults, demonstrates limited clinical utility of its standard first-line R-CHOP regimen due to cumulative cardiotoxicity from anthracycline doxorubicin (DOX). This is particularly impactful for elderly patients or those with cardiac comorbidities. The irreversible myocardial damage can significantly compromise long-term quality of life even after disease remission. Studies indicate a 15.7% incidence of congestive heart failure at cumulative DOX doses of 500 mg/m^2^, directly correlating with its dose-limiting threshold (450–550 mg/m^2^) (18, 19). Thus, mitigating anthracycline cardiotoxicity while maintaining efficacy has become pivotal for improving DLBCL outcomes. In this context, pegylated liposomal doxorubicin (PLD) demonstrates distinct advantages through unique pharmacokinetics: Liposomal encapsulation elevates the area under the concentration-time curve (AUC) by approximately 300-fold (a conservative estimate, with actual values ranging from 300–2,800-fold depending on dose and study) compared with conventional doxorubicin (9), and enhances tumor drug concentration by 20–60-fold vs. normal tissue (20, 21), while elevating the cumulative dose threshold for 5% cardiotoxicity to 1,350 mg/m^2^ (9). The 100 nm particles leverage enhanced permeability and retention (EPR) effects for passive targeting, prolonging the circulation half-life (PLD: 55–75 h for typical clinical doses) in contrast to conventional DOX with a terminal phase half-life of 20–48 h (per FDA label) (9). Notably, PLD's half-life reflects the clearance of intact liposomes from the vascular space, while DOX's terminal half-life represents slow drug release from deeply bound tissue compartments—these are fundamentally distinct pharmacokinetic processes that require explicit acknowledgment in direct comparison. Moreover, >93%−99% of circulating doxorubicin after PLD administration remains liposome-encapsulated, resulting in a 10–30-fold lower peak free drug concentration (Cmax) compared with conventional DOX (9), which is critical for mitigating cardiotoxicity. Compared to DOX, PLD maintains antitumor efficacy while significantly reducing cardiotoxicity, alopecia, and myelosuppression (21), with its common toxicities including mild myelosuppression (e.g., neutropenia), gastrointestinal reactions, anemia, and hepatic/renal dysfunction (22). The robust cardioprotective effects of PLD are underpinned by four convergent mechanisms: reduced free doxorubicin Cmax mimicking continuous infusion, physical blockage of liposome extravasation by the intact cardiac endothelium, documented lower myocardial drug concentrations in preclinical models, and clinically validated lower cardiotoxicity risk in randomized trials (9).

Notably, the fundamentally distinct pharmacokinetic profiles of PLD (circulation half-life: 55–75 h) and conventional DOX (terminal phase half-life: 20–48 h), coupled with the lack of formal pharmacokinetic, pharmacodynamic, or clinical dose-finding studies, have precluded the establishment of reliable dose conversion algorithms, resulting in insufficient dose optimization research for PLD in DLBCL (9). A critical caveat is that no published study has formally validated the clinical equivalence of PLD 30 mg/m^2^ to DOX 50 mg/m^2^ in any tumor type, including lymphoma; clinical evidence even suggests PLD 30 mg/m^2^ may be subtherapeutic, while PLD 40 mg/m^2^ more closely approximates the efficacy of standard R-CHOP (DOX 50 mg/m^2^) (23, 24). Current recommended PLD doses (20–45 mg/m^2^) derive primarily from single-arm trials or historical controls (20, 23–25), lacking rigorous dose-escalation studies or efficacy-toxicity tradeoff analyses; additionally, neither NCCN nor ESMO guidelines specifically recommend PLD as a standard substitute for DOX in R-CHOP for DLBCL. It is also essential to distinguish PLD (Caelyx/Doxil) from non-pegylated liposomal doxorubicin (NPLD/Myocet), as the existing evidence base for liposomal doxorubicin in DLBCL predominantly uses NPLD at a 1:1 dose substitution ratio—these are pharmacologically distinct formulations and cannot be conflated (9). Alternative regimens like epirubicin-based R-CEOP70/90 (26, 27) or pirarubicin (50 mg/m^2^) (28–30) demonstrate comparable efficacy to standard R-CHOP (doxorubicin 50 mg/m^2^), yet fail to circumvent the intrinsic toxicities of anthracyclines. PLD's extended activity presents an interesting contrast to dose-adjusted R-EPOCH (DA-EPOCH-R), an intensive regimen that achieves prolonged drug exposure through 96-h continuous infusion of doxorubicin, etoposide, and vincristine. While DA-EPOCH-R has demonstrated efficacy in high-risk DLBCL subtypes, it is associated with substantial toxicity (neutropenic fever: 13%−33%; hospitalization: 16%−35%) and logistical challenges that limit its widespread adoption (31). Both approaches share the biological rationale of sustained anthracycline exposure—PLD through its slow-release liposomal pharmacokinetics and DA-EPOCH-R through prolonged infusion—yet PLD offers this advantage with a more favorable safety profile and greater practical convenience (31). This sustained exposure benefit may optimize treatment not only for elderly patients or those with cardiac risk factors but also for the general DLBCL population. However, existing studies on PLD in DLBCL have predominantly employed single-arm designs with limited sample sizes and focused on elderly populations [e.g., Avilés et al. (32): 20 treatment-naïve DLBCL cases; Zaja et al. (20): 30 elderly DLBCL patients; Schmitt et al. (21): 21 NHL patients with cardiac risk factors; Oki et al. (23): 80 elderly DLBCL cases], collectively demonstrating a paucity of systematic evaluation of PLD in broader patient populations.

This propensity score-matched (PSM) analysis of 512 DLBCL patients (aged 18–82 years) compared the efficacy and safety of R-CDOP vs. R-CHOP, while evaluating PLD's applicability and dose-response relationship in general populations. While not reaching statistical significance, the HIGH-PLD [29.5 (25.5–40) mg/m^2^] showed numerically improved PFS outcomes compared to both the DOX group [46.2 (20–50) mg/m^2^; 2-year PFS: 81.0% vs. 70.5%, *P* = 0.354, Holm-Bonferroni *P* = 0.707] and the STD-DOX [49.0 (40–50) mg/m^2^; 2-year PFS: 81.0% vs. 69.4%, *P* = 0.349, Holm-Bonferroni *P* = 0.697], suggesting a potential clinical benefit. Notably, the HIGH-PLD regimen demonstrated statistically significant PFS superiority over the LOW-DOX [32.4 (20–40) mg/m^2^; 79.9% vs. 59.8%, *P* = 0.0066, Holm-Bonferroni *P* = 0.0132]. This dose-dependent efficacy likely stems from PLD's liposomal structure, which enhances tumor targeting via the enhanced permeability and retention (EPR) effect and prolongs half-life, thereby increasing intratumoral drug concentration while reducing systemic toxicity (9). Although HIGH-PLD showed favorable OS trends (*P* > 0.05), statistical significance was not achieved, potentially due to limited sample size or follow-up duration. LOW-PLD [21.5 (5–25.5) mg/m^2^] showed comparable PFS and OS to all DOX groups (*P* > 0.05). Mechanistically, higher PLD doses sustain therapeutic tumor concentrations through liposomal drug release, whereas lower doses may fail to overcome tumor microenvironment barriers (33). These findings align with Li et al. (24) (*n* = 335 elderly DLBCL), where higher PLD doses [29.5 (25.5–45) mg/m^2^] improved survival outcomes while lower doses prioritized safety at potential efficacy cost. Collectively, these results underscore PLD's dose-intensity dependence and the need for personalized dosing based on patient characteristics. It is important to clarify our rationale for excluding R-miniCHOP while retaining patients with reduced doxorubicin doses within standard R-CHOP. R-miniCHOP is a pre-defined attenuated regimen with systematic dose reductions across all cytotoxic agents (typically vincristine 1 mg fixed dose, doxorubicin 25 mg/m^2^, cyclophosphamide 400 mg/m^2^, and prednisone 40 mg/m^2^ × 5 days), specifically designed for frail elderly patients deemed unable to tolerate full-dose chemotherapy. In contrast, patients in our LOW-DOX subgroup received standard R-CHOP with only doxorubicin dose modifications, while maintaining standard doses of other agents. This individualized approach reflects real-world clinical decision-making where physicians adjust anthracycline exposure based on patient-specific factors while preserving overall treatment intensity. Including these patients allows evaluation of dose-response relationships within the R-CHOP framework, which has direct clinical relevance for treatment optimization. Regarding the LOW-DOX subgroup, potential reasons for dose reduction within the standard R-CHOP framework include: (1) advanced age with borderline fitness criteria; (2) mild pre-existing comorbidities not meeting exclusion thresholds; (3) early-cycle toxicities prompting subsequent dose adjustments; and (4) physician preference based on individual risk-benefit assessment. These patients represent a clinically relevant population frequently encountered in real-world practice, where strict adherence to protocol-specified doses is often modified based on clinical judgment. An important consideration when interpreting the outcomes of the LOW-DOX subgroup is the confounding effect of patient frailty. Dose reductions in this group were predominantly driven by advanced age (36.2% aged ≥70 years), poor performance status (38.3% ECOG ≥2), and baseline cytopenias rather than cardiac dysfunction (all baseline LVEF ≥60%). These factors are independently associated with inferior survival outcomes regardless of anthracycline dose intensity. Although our PSM model adjusted for age, ECOG performance status, and other measured prognostic factors, residual confounding by unmeasured dimensions of frailty—such as comorbidity burden, nutritional status, and functional reserve—cannot be fully excluded. Within the LOW-DOX subgroup, patients with the most adverse clinical profiles (age ≥60 and ECOG ≥2) had a median OS of 3.1 years compared with 4.6 years for those without either risk factor, further supporting the contribution of patient vulnerability to the observed outcomes. Consequently, the survival patterns in the LOW-DOX subgroup should be interpreted as reflecting the combined effect of patient frailty and reduced dose intensity, rather than dose intensity alone. An important methodological consideration is that the dose subgroups in this study are subject to confounding by indication. Patients receiving reduced anthracycline doses (LOW-DOX) were more likely to be elderly, have poor performance status, and harbor high-risk disease features, reflecting clinical decisions to attenuate treatment intensity in frailer patients. This systematic treatment allocation pattern means that the observed baseline differences between subgroups are not random confounders that can be fully eliminated through PSM, but rather reflect the clinical reality of individualized dosing. Our pairwise PSM approach, combined with Holm-Bonferroni correction and sensitivity analyses incorporating LVEF, represents the most appropriate strategy to address these inherent biases while preserving the ability to answer clinically actionable pairwise questions.

Clinical management of elderly DLBCL patients presents a therapeutic dilemma regarding anthracycline dosing: while dose-reduced R-miniCHOP improves tolerability, its potential efficacy compromise remains concerning. Merli et al. (34) reported reduced mortality (HR = 0.13, *P* = 0.011) with R-miniCHOP in low-risk patients >72 years, whereas Al-Sarayfi et al. (35) demonstrated inferior 2-year PFS (51% vs. 68%, *P* < 0.01) and OS (60% vs. 75%, *P* < 0.01) vs. standard dosing in ≥65-year-olds, with 73% increased mortality risk (HR = 1.73). The central challenge lies in determining whether dose reduction's safety benefits outweigh survival losses from diminished treatment response. The PLD-based R-CDOP regimen addresses this by modifying drug delivery systems rather than merely reducing doses. Our elderly subgroup analysis (≥60 years) revealed non-significant but favorable survival trends for PLD: 10.3% higher 2-year PFS (68.1% vs. 57.80%, *P* = 0.683, Holm-Bonferroni *P* = 1) and 0.2% improved OS (72.6% vs. 72.4%, *P* = 0.91, Holm-Bonferroni *P* = 1). This may reflect PLD's enhanced tolerability and reduced toxicity, potentially offering dual benefits for elderly patients.

Regarding safety, PLD demonstrated significantly lower rates of hematologic toxicity and hepatic dysfunction vs. DOX (all *P* < 0.05), consistent with findings by Oki et al. (23). This advantage likely stems from PLD's pegylated liposomal structure minimizing free drug exposure to bone marrow and liver tissue (22). Although cardiac toxicity was numerically lower with PLD (17.6% vs. 24.6%, *P* = 0.197), the difference lacked statistical significance, potentially because: the 24-month median follow-up may underestimate PLD's long-term cardioprotection, as evidenced by metastatic breast cancer trials showing stronger cardioprotection after 5+ years (HR = 3.16, *P* < 0.001) (22); Baseline cardiac risk imbalances (e.g., hypertension/diabetes) may obscure true benefits, requiring subgroup analyses. Additionally, pneumonia incidence was marginally higher with PLD (21.8% vs. 19.4%, *P* = 0.639), though not statistically significant. A recognized limitation of PLD is increased infection risk, potentially due to liposomal pulmonary accumulation causing oxidative stress and immune dysregulation (36). Prophylactic strategies include trimethoprim-sulfamethoxazole or N-acetylcysteine to mitigate opportunistic infections (37). For high-risk patients, clinicians must balance PLD's cardioprotective benefits against pulmonary toxicity, considering dose modifications or optimized combination regimens when warranted, alongside enhanced oxygenation monitoring and radiographic surveillance during treatment.

## Limitations

While this retrospective analysis yields valuable clinical insights, it has inherent limitations, including potential selection bias in cohort enrollment. Although propensity score matching (PSM) balanced baseline characteristics between groups, unmeasured or unmatched confounders (e.g., GCB subtype) may still have influenced study outcomes. Furthermore, post-matching standardized mean differences (SMD) for some covariates exceeded the recommended threshold (< 0.1), indicating potential residual confounding bias. Additionally, the relatively short median follow-up duration (33 months) precluded the assessment of long-term cardiac function and late-onset cardiotoxicity—an adverse event that typically manifests years after anthracycline exposure. Given this limitation, we propose a structured long-term cardiac monitoring plan for DLBCL patients receiving anthracycline-based therapy to address the unmet need for long-term cardiac safety surveillance: transthoracic echocardiography (TTE) to assess LVEF at 1 year, 3 years, 5 years, and 10 years after treatment initiation, with more frequent monitoring (e.g., every 6–12 months in the first 3 years) for high-risk patients (e.g., elderly patients ≥60 years, those with pre-existing cardiac comorbidities such as hypertension or diabetes, or cumulative anthracycline doses >300 mg/m^2^). Despite these limitations, the study provides real-world evidence for patient populations commonly excluded from clinical trials (e.g., those with cardiac comorbidities or immunocompromised status). Future investigations with larger cohorts and extended follow-up are needed to validate the observed survival benefits, establish more comprehensive risk stratification strategies, and compare the long-term benefit-risk profiles of PLD and DOX in high-risk populations.

Several key methodological limitations also merit consideration. First, baseline left ventricular ejection fraction (LVEF) data were available for only 68.4% of patients (350/512). While LVEF was not included in the primary PSM model due to incomplete data availability, a comparison of baseline characteristics between patients with and without measurable LVEF data revealed no clinically meaningful differences (all SMD < 0.15 except for extranodal involvement, SMD = 0.20), supporting a missing-at-random (MAR) pattern for the missing data. Of note, patients with severe cardiac dysfunction (NYHA class ≥ III, Child-Pugh grade ≥ B, or eGFR < 30 mL/min/1.73 m^2^) were excluded at enrollment, which ensured that all study participants had adequate baseline cardiac function—consistent with the observation that all evaluable patients had baseline LVEF ≥ 60%—and further supports that LVEF was unlikely to be a major confounder in this cohort. A pre-planned sensitivity analysis that incorporated LVEF as an additional matching covariate yielded results consistent with the primary analysis, confirming that baseline cardiac function did not confound the observed treatment effects. Second, pairwise rather than simultaneous four-group PSM was employed in this study. Although four-group generalized propensity score methods are available, their application was not feasible in our cohort due to the small sample size of the LOW-DOX subgroup (*n* = 47), a limited common support region resulting from confounding by indication, and the inherently pairwise nature of the key clinical research questions. We mitigated concerns related to multiple testing via Holm-Bonferroni correction, and the consistency of study results across all analytical approaches (pairwise PSM, LVEF-adjusted sensitivity analysis, and multivariable Cox regression) validates the robustness of our conclusions. Third, the reasons for doxorubicin dose reduction were ascertained retrospectively and likely reflect a combination of age-related frailty, comorbidity burden, and clinician judgment. The inferior outcomes observed in the LOW-DOX subgroup cannot be attributed solely to reduced dose intensity, as residual confounding by unmeasured frailty indicators likely persists despite PSM adjustment. Fourth, the limited number of outcome events in the smaller subgroups [e.g., 12 overall survival (OS) events in the HIGH-PLD subgroup, 10 in the LOW-PLD subgroup] limits the statistical power to detect modest treatment effects—an inherent constraint of the retrospective study design—and reduces the reliability of multivariable adjustment. This underscores the hypothesis-generating nature of the present study, with its findings requiring further confirmation in larger prospective cohorts.

In conclusion, Pegylated liposomal doxorubicin (PLD) achieves an optimized efficacy-safety balance in DLBCL treatment through unique pharmacokinetic properties. Our study demonstrates that all PLD groups [25.5 (5–40) mg/m^2^] shows comparable efficacy to standard doxorubicin [49.0 (40–50) mg/m^2^] in progression-free survival (PFS, *P* > 0.05) and overall survival (OS, *P* > 0.05). HIGH-PLD achieved significantly superior PFS vs. low-dose DOX. Safety analyses reveal significantly reduced hematologic toxicities and hepatic dysfunction (*P* < 0.05), with a trend toward lower cardiotoxicity. However, the higher pneumonia incidence with PLD warrants clinical vigilance. The elderly subgroup (≥60 years) derived clinical benefits consistent with the overall population. Therefore, clinical implementation requires comprehensive benefit-risk assessment of PLD. Future studies should optimize PLD dosing strategies and explore applications across diverse patient subgroups, while enhancing monitoring for pulmonary toxicity risks, to better balance therapeutic efficacy and safety for precision DLBCL management.

## Data Availability

The raw data supporting the conclusions of this article will be made available by the authors, without undue reservation.
