# Supplementary material for: Propensity-matched study of liposomal doxorubicin vs. doxorubicin in first-line DLBCL treatment: efficacy and safety
Source: Front Med (Lausanne). 2026 Apr 1;13:1769270. doi: 10.3389/fmed.2026.1769270 (PMC13079127; doi:10.3389/fmed.2026.1769270)
Supplement: Supplementary file 1 [file Table_1.docx]

|  |  |  | | Original queue | | | |  |  |  | 1:2 matching queue | | |  |  |
| --- | --- | --- | --- | --- | --- | --- | --- | --- | --- | --- | --- | --- | --- | --- | --- |
|  | DOX | | % | | HIGH-PLD | % | P | SMD |  | DOX | % HIGH-PLD | | % | P | SMD |
| N | 370 | |  | | 71 |  |  |  |  | 142 |  | 71 |  |  |  |
| Male sex | 206 | | (55.7) | | 31 | (43.7) | 0.084 | 0.242 |  | 82 | (57.7) | 31 | (43.7) | 0.072 | 0.285 |
| >60 years |  | |  | |  |  |  |  |  |  |  |  |  |  |  |
| No | 255 | | (68.9) | | 43 | (60.6) | 0.215 | 0.176 |  | 87 | (61.3) | 43 | (60.6) | 1 | 0.014 |
| Yes | 115 | | (31.1) | | 28 | (39.4) |  |  |  | 55 | (38.7) | 28 | (39.4) |  |  |
| Gene Expression Profiling |  | |  | |  |  |  |  |  |  |  |  |  |  |  |
| GCB | 207 | | (55.9) | | 39 | (54.9) | 0.969 | 0.025 |  | 77 | (54.2) | 39 | (54.9) | 1 | 0.014 |
| non-GCB | 121 | | (32.7) | | 24 | (33.8) |  |  |  | 46 | (32.4) | 24 | (33.8) |  |  |
| Unknown | 42 | | （11.4） | | 8 | （11.3） |  |  |  | 19 | （13.4） | 8 | （11.3） |  |  |
| Lactate dehydrogenase |  | |  | |  |  |  |  |  |  |  |  |  |  |  |
| Normal | 192 | | (51.9) | | 43 | (60.6) | 0.226 | 0.175 |  | 82 | (57.7) | 43 | (60.6) | 0.806 | 0.057 |
| Elevated | 178 | | (48.1) | | 28 | (39.4) |  |  |  | 60 | (42.3) | 28 | (39.4) |  |  |
| Lugano stage |  | |  | |  |  |  |  |  |  |  |  |  |  |  |
| I-II | 165 | | (44.6) | | 30 | (42.3) | 0.815 | 0.047 |  | 52 | (36.6) | 30 | (42.3) | 0.517 | 0.115 |
| III-IV | 205 | | (55.4) | | 41 | (57.7) |  |  |  | 90 | (63.4) | 41 | (57.7) |  |  |
| Number of extranodal sites |  | |  | |  |  |  |  |  |  |  |  |  |  |  |
| 0-1 | 293 | | (79.2) | | 51 | (71.8) | 0.224 | 0.172 |  | 102 | (71.1) | 51 | (71.8) | 1 | 0.001 |
| >2 | 77 | | (20.8) | | 20 | (28.2) |  |  |  | 40 | (28.9) | 20 | (28.2) |  |  |
| ECOG |  | |  | |  |  |  |  |  |  |  |  |  |  |  |
| 0-1 | 286 | | (77.3) | | 49 | (69.0) | 0.179 | 0.188 |  | 94 | (66.2) | 49 | (69.0) | 0.797 | 0.06 |
| 2-5 | 84 | | (22.7) | | 22 | (31.0) |  |  |  | 48 | (33.8) | 22 | (31.0) |  |  |

**Table S1．Baseline data of the DOX group and the HIGH-PLD group before and after PSM 1:2 matching, n(%).** Abbreviations: DOX（Doxorubicin group），HIGH-PLD（high-dose PLD subgroup）, SMD（Standardized Mean Difference）, ECOG（Eastern Cooperative Oncology Group）, GCB（germinal center B-cell）. Original queue: Pre-matching baseline characteristics of the DOX and HIGH-PLD groups. 1:2 matched queue: Post-matching characteristics after 1:2 PSM adjusting for covariates (age, LDH, Lugano stage, extranodal involvement, ECOG). Notes: No significant differences pre- or post-matching (P>0.05). Post-matching SMD<0.1 for age (>60 years), sex, LDH, extranodal involvement, and ECOG; SMD <0.2 for Lugano stage.
