# Supplementary material for: Propensity-matched study of liposomal doxorubicin vs. doxorubicin in first-line DLBCL treatment: efficacy and safety
Source: Front Med (Lausanne). 2026 Apr 1;13:1769270. doi: 10.3389/fmed.2026.1769270 (PMC13079127; doi:10.3389/fmed.2026.1769270)
Supplement: Supplementary file 2 [file Table_2.docx]

|  |  |  | | Original queue | | | |  |  |  | 1:2 matching queue | | |  |  |
| --- | --- | --- | --- | --- | --- | --- | --- | --- | --- | --- | --- | --- | --- | --- | --- |
|  | DOX | | % | | LOW-PLD | % | P | SMD |  | DOX | % | LOW-PLD | % | P | SMD |
| N | 370 | |  | | 71 |  |  |  |  | 142 |  | 71 |  |  |  |
| Male sex | 206 | | (55.7) | | 45 | (63.4) | 0.285 | 0.157 |  | 78 | (54.9) | 45 | (63.4) | 0.303 | 0.173 |
| >60 years |  | |  | |  |  |  |  |  |  |  |  |  |  |  |
| No | 255 | | (68.9) | | 35 | (49.3) | 0.002 | 0.407 |  | 69 | (48.6) | 35 | (49.3) | 1 | 0.014 |
| Yes | 115 | | (31.1) | | 36 | (50.7) |  |  |  | 73 | (51.4) | 36 | (50.7) |  |  |
| Gene Expression Profiling |  | |  | |  |  |  |  |  |  |  |  |  |  |  |
| GCB | 207 | | （55.9) | | 33 | (46.5) | 0.236 | 0.184 |  | 83 | (58.4) | 33 | (46.5) | 0.208 | 0.22 |
| non-GCB | 121 | | (32.7) | | 28 | (39.4) |  |  |  | 45 | (31.7) | 28 | (39.4) |  |  |
| Unknown | 42 | | (11.4) | | 10 | (14.1) |  |  |  | 14 | (9.9) | 10 | (14.1) |  |  |
| Lactate dehydrogenase |  | |  | |  |  |  |  |  |  |  |  |  |  |  |
| Normal | 192 | | (51.9) | | 40 | (56.3) | 0.577 | 0.089 |  | 83 | (58.5) | 40 | (56.3) | 0.883 | 0.043 |
| Elevated | 178 | | (48.1) | | 31 | (43.7) |  |  |  | 59 | (41.5) | 31 | (43.7) |  |  |
| Lugano stage |  | |  | |  |  |  |  |  |  |  |  |  |  |  |
| I-II | 165 | | (44.6) | | 28 | (39.4) | 0.502 | 0.105 |  | 57 | (40.1) | 28 | (39.4) | 1 | 0.014 |
| III-IV | 205 | | (55.4) | | 43 | (60.6) |  |  |  | 85 | (59.9) | 43 | (60.6) |  |  |
| Number of extranodal sites |  | |  | |  |  |  |  |  |  |  |  |  |  |  |
| 0-1 | 293 | | (79.2) | | 50 | (70.4) | 0.141 | 0.203 |  | 103 | (72.5) | 50 | (70.4) | 0.872 | 0.042 |
| >2 | 77 | | (20.8) | | 21 | (29.6) |  |  |  | 39 | (27.5) | 21 | (29.6) |  |  |
| ECOG |  | |  | |  |  |  |  |  |  |  |  |  |  |  |
| 0-1 | 286 | | (77.3) | | 45 | (63.4) | 0.02 | 0.308 |  | 93 | (65.5) | 45 | (63.4) | 0.879 | 0.044 |
| 2-5 | 84 | | (22.7) | | 26 | (36.6) |  |  |  | 49 | (34.5) | 26 | (36.6) |  |  |

**Table S2．Baseline data of the DOX group and the LOW-PLD group before and after PSM 1:2 matching, n(%).** Abbreviations: DOX（Doxorubicin group），LOW-PLD（low-dose PLD subgroup）, SMD（Standardized Mean Difference）, ECOG（Eastern Cooperative Oncology Group）, GCB（germinal center B-cell）. Original queue: Pre-matching baseline characteristics of the DOX and LOW-PLD groups. 1:2 matched queue: Post-matching characteristics after 1:2 PSM adjusting for covariates (age, LDH, Lugano stage, extranodal involvement, ECOG). Notes: Pre-matching significant differences in age (>60 years) and ECOG (P<0.05); post-matching balance achieved (P>0.05). Post-matching SMD<0.1 for age, sex, LDH, Lugano stage, extranodal involvement, and ECOG.
