# Supplementary material for: Propensity-matched study of liposomal doxorubicin vs. doxorubicin in first-line DLBCL treatment: efficacy and safety
Source: Front Med (Lausanne). 2026 Apr 1;13:1769270. doi: 10.3389/fmed.2026.1769270 (PMC13079127; doi:10.3389/fmed.2026.1769270)
Supplement: Supplementary file 3 [file Table_3.docx]

|  |  |  | | Original queue | | | |  |  |  | 1:2 matching queue | | |  |  |
| --- | --- | --- | --- | --- | --- | --- | --- | --- | --- | --- | --- | --- | --- | --- | --- |
|  | STD-DOX | | % | | HIGH-PLD | % | P | SMD |  | STD-DOX | % | HIGH-PLD | % | P | SMD |
| N | 323 | |  | | 71 |  |  |  |  | 142 |  | 71 |  |  |  |
| Male sex | 180 | | (55.7) | | 31 | (43.7) | 0.086 | 0.243 |  | 85 | (59.9) | 31 | (43.7) | 0.036 | 0.328 |
| >60 years |  | |  | |  |  |  |  |  |  |  |  |  |  |  |
| No | 233 | | (72.1) | | 43 | (60.6) | 0.074 | 0.247 |  | 93 | (65.5) | 43 | (60.6) | 0.579 | 0.102 |
| Yes | 90 | | (27.9) | | 28 | (39.4) |  |  |  | 49 | (34.5) | 28 | (39.4) |  |  |
| Gene Expression Profiling |  | |  | |  |  |  |  |  |  |  |  |  |  |  |
| GCB | 181 | | (56.0) | | 39 | (54.9) | 0.925 | 0.033 |  | 83 | (58.5) | 39 | (54.9) | 0.707 | 0.083 |
| non-GCB | 104 | | (32.2) | | 24 | (33.8) |  |  |  | 43 | (30.3) | 24 | (33.8) |  |  |
| Unknown | 38 | | (11.8) | | 8 | （11.3） |  |  |  | 16 | (11.2) | 8 | （11.3） |  |  |
| Lactate dehydrogenase |  | |  | |  |  |  |  |  |  |  |  |  |  |  |
| Normal | 174 | | (53.9) | | 43 | (60.6) | 0.371 | 0.136 |  | 77 | (54.2) | 43 | (60.6) | 0.464 | 0.128 |
| Elevated | 149 | | (46.1) | | 28 | (39.4) |  |  |  | 65 | (45.8) | 28 | (39.4) |  |  |
| Lugano stage |  | |  | |  |  |  |  |  |  |  |  |  |  |  |
| I-II | 147 | | (45.5) | | 30 | (42.3) | 0.713 | 0.066 |  | 52 | (36.6) | 30 | (42.3) | 0.517 | 0.115 |
| III-IV | 176 | | (54.5) | | 41 | (57.7) |  |  |  | 90 | (63.4) | 41 | (57.7) |  |  |
| Number of extranodal sites |  | |  | |  |  |  |  |  |  |  |  |  |  |  |
| 0-1 | 264 | | (81.7) | | 51 | (71.8) | 0.085 | 0.236 |  | 96 | (67.6) | 51 | (71.8) | 0.637 | 0.092 |
| >2 | 59 | | (18.3) | | 20 | (28.2) |  |  |  | 46 | (32.4) | 20 | (28.2) |  |  |
| ECOG |  | |  | |  |  |  |  |  |  |  |  |  |  |  |
| 0-1 | 257 | | (79.6) | | 49 | (69.0) | 0.076 | 0.243 |  | 95 | (65.5) | 49 | (69.0) | 0.877 | 0.045 |
| 2-5 | 66 | | (20.4) | | 22 | (31.0) |  |  |  | 47 | (34.5) | 22 | (31.0) |  |  |

**Table S3．Baseline data of the STD-DOX group and the HIGH-PLD group before and after PSM 1:2 matching, n(%).**Abbreviations: STD-DOX (standard-dose DOX subgroup), HIGH-PLD (high-dose PLD subgroup), SMD (Standardized Mean Difference), ECOG (Eastern Cooperative Oncology Group), GCB (germinal center B-cell).Original queue: Pre-matching baseline characteristics of STD-DOX and HIGH-PLD groups.1:2 matched queue: Post-matching characteristics after 1:2 PSM adjusting for covariates (age (>60 years), LDH, Lugano stage, Number of extranodal sites , ECOG).Notes: No significant differences pre- or post-matching (P> 0.05). Post-matching SMD <0.1 for Number of extranodal sites and ECOG; SMD <0.2 for age (>60 years), LDH, and Lugano stage.
