# Supplementary material for: Propensity-matched study of liposomal doxorubicin vs. doxorubicin in first-line DLBCL treatment: efficacy and safety
Source: Front Med (Lausanne). 2026 Apr 1;13:1769270. doi: 10.3389/fmed.2026.1769270 (PMC13079127; doi:10.3389/fmed.2026.1769270)
Supplement: Supplementary file 4 [file Table_4.docx]

|  |  |  | | Original queue | | | |  |  |  | 1:2 matching queue | | |  |  |
| --- | --- | --- | --- | --- | --- | --- | --- | --- | --- | --- | --- | --- | --- | --- | --- |
|  | STD-DOX | | % | | LOW-PLD | % | P | SMD |  | STD-DOX | % | LOW-PLD | % | P | SMD |
| N | 323 | |  | | 71 |  |  |  |  | 142 |  | 71 |  |  |  |
| Male sex | 180 | | (55.7) | | 45 | (63.4) | 0.295 | 0.156 |  | 82 | (57.7) | 45 | (63.4) | 0.521 | 0.115 |
| >60 years |  | |  | |  |  |  |  |  |  |  |  |  |  |  |
| No | 233 | | (72.1) | | 35 | (49.3) | <0.001 | 0.481 |  | 70 | (49.3) | 35 | (49.3) | 1 | <0.001 |
| Yes | 90 | | (27.9) | | 36 | (50.7) |  |  |  | 72 | (50.7) | 36 | (50.7) |  |  |
| Gene Expression Profiling |  | |  | |  |  |  |  |  |  |  |  |  |  |  |
| GCB | 181 | | (56.0) | | 33 | (46.5) | 0.219 | 0.192 |  | 78 | (54.9) | 33 | (46.5) | 0.355 | 0.169 |
| non-GCB | 104 | | (32.2) | | 28 | (39.4) |  |  |  | 47 | (33.1) | 28 | (39.4) |  |  |
| Unknown | 38 | | (11.8) | | 10 | (14.1) |  |  |  | 17 | (12.0) | 10 | (14.1) |  |  |
| Lactate dehydrogenase |  | |  | |  |  |  |  |  |  |  |  |  |  |  |
| Normal | 174 | | (53.9) | | 40 | (56.3) | 0.805 | 0.05 |  | 89 | (62.7) | 40 | (56.3) | 0.457 | 0.129 |
| Elevated | 149 | | (46.1) | | 31 | (43.7) |  |  |  | 53 | (37.3) | 31 | (43.7) |  |  |
| Lugano stage |  | |  | |  |  |  |  |  |  |  |  |  |  |  |
| I-II | 147 | | (45.5) | | 28 | (39.4) | 0.423 | 0.123 |  | 63 | (44.4) | 28 | (39.4) | 0.59 | 0.1 |
| III-IV | 176 | | (54.5) | | 43 | (60.6) |  |  |  | 79 | (55.6) | 43 | (60.6) |  |  |
| Number of extranodal sites |  | |  | |  |  |  |  |  |  |  |  |  |  |  |
| 0-1 | 264 | | (81.7) | | 50 | (70.4) | 0.047 | 0.268 |  | 108 | (76.1) | 50 | (70.4) | 0.472 | 0.128 |
| >2 | 59 | | (18.3) | | 21 | (29.6) |  |  |  | 34 | (23.9) | 21 | (29.6) |  |  |
| ECOG |  | |  | |  |  |  |  |  |  |  |  |  |  |  |
| 0-1 | 257 | | (79.6) | | 45 | (63.4) | 0.006 | 0.364 |  | 102 | (71.8) | 45 | (63.4) | 0.271 | 0.181 |
| 2-5 | 66 | | (20.4) | | 26 | (36.6) |  |  |  | 40 | (28.2) | 26 | (36.6) |  |  |

**Table S4．Baseline data of the STD-DOX group and the LOW-PLD group before and after PSM 1:2 matching, n(%).**Abbreviations: STD-DOX (standard-dose DOX subgroup), LOW-PLD (low-dose PLD subgroup), SMD (Standardized Mean Difference), ECOG (Eastern Cooperative Oncology Group), GCB (germinal center B-cell).Original queue: Pre-matching baseline characteristics of STD-DOX and LOW-PLD groups.1:2 matched queue: Post-matching characteristics after 1:2 PSM adjusting for covariates (age (>60 years), LDH, Lugano stage, Number of extranodal sites , ECOG).Notes: Pre-matching significant differences in age (>60 years), Number of extranodal sites , and ECOG (P<0.05); post-matching balance achieved (P>0.05). Post-matching SMD<0.1 for age (>60 years) and Lugano stage; SMD<0.2 for LDH, Number of extranodal sites and ECOG.
