# Supplementary material for: Propensity-matched study of liposomal doxorubicin vs. doxorubicin in first-line DLBCL treatment: efficacy and safety
Source: Front Med (Lausanne). 2026 Apr 1;13:1769270. doi: 10.3389/fmed.2026.1769270 (PMC13079127; doi:10.3389/fmed.2026.1769270)
Supplement: Supplementary file 5 [file Table_5.docx]

|  |  |  | | Original queue | | | |  |  |  | 1:2 matching queue | | |  |  |
| --- | --- | --- | --- | --- | --- | --- | --- | --- | --- | --- | --- | --- | --- | --- | --- |
|  | LOW-DOX | | % | | HIGH-PLD | % | P | SMD |  | LOW-DOX | % | HIGH-PLD | % | P | SMD |
| N | 47 | |  | | 71 |  |  |  |  | 47 |  | 47 |  |  |  |
| Male sex | 26 | | (55.3) | | 31 | (43.7) | 0.293 | 0.235 |  | 26 | (55.3) | 19 | (40.4) | 0.215 | 0.302 |
| >60 years |  | |  | |  |  |  |  |  |  |  |  |  |  |  |
| No | 22 | | (46.8) | | 43 | (60.6) | 0.2 | 0.279 |  | 22 | (46.8) | 26 | (55.3) | 0.536 | 0.171 |
| Yes | 25 | | (53.2) | | 28 | (39.4) |  |  |  | 25 | (53.2) | 21 | (44.7) |  |  |
| Gene Expression Profiling |  | |  | |  |  |  |  |  |  |  |  |  |  |  |
| GCB | 26 | | (55.3) | | 39 | (54.9) | 1 | 0.03 |  | 26 | (55.3) | 26 | (55.3) | 0.957 | 0.061 |
| non-GCB | 17 | | (36.2) | | 24 | (33.8) |  |  |  | 17 | (36.2) | 15 | (31.9) |  |  |
| Unknown | 4 | | (8.5) | | 8 | （11.3） |  |  |  | 4 | (8.5) | 6 | (12.8) |  |  |
| Lactate dehydrogenase |  | |  | |  |  |  |  |  |  |  |  |  |  |  |
| Normal | 18 | | (38.3) | | 43 | (60.6) | 0.029 | 0.457 |  | 18 | (38.3) | 20 | (42.6) | 0.834 | 0.087 |
| Elevated | 29 | | (61.7) | | 28 | (39.4) |  |  |  | 29 | (61.7) | 27 | (57.4) |  |  |
| Lugano stage |  | |  | |  |  |  |  |  |  |  |  |  |  |  |
| I-II | 18 | | (38.3) | | 30 | (42.3) | 0.813 | 0.081 |  | 18 | (38.3) | 19 | (40.4) | 1 | 0.044 |
| III-IV | 29 | | (61.7) | | 41 | (57.7) |  |  |  | 29 | (61.7) | 28 | (59.6) |  |  |
| Number of extranodal sites |  | |  | |  |  |  |  |  |  |  |  |  |  |  |
| 0-1 | 29 | | (61.7) | | 51 | (71.8) | 0.341 | 0.216 |  | 29 | (61.7) | 30 | (63.8) | 1 | 0.044 |
| >2 | 18 | | (38.3) | | 20 | (28.2) |  |  |  | 18 | (38.3) | 17 | (36.2) |  |  |
| ECOG |  | |  | |  |  |  |  |  |  |  |  |  |  |  |
| 0-1 | 29 | | (61.7) | | 49 | (69.0) | 0.533 | 0.154 |  | 29 | (61.7) | 30 | (63.8) | 1 | 0.044 |
| 2-5 | 18 | | (38.3) | | 22 | (31.0) |  |  |  | 18 | (38.3) | 17 | (36.2) |  |  |

**Table S5．Baseline data of the LOW-DOX group and the HIGH-PLD group before and after PSM 1:1 matching, n(%).** Abbreviations: LOW-DOX（low-dose DOX subgroup），HIGH-PLD（high-dose PLD subgroup）, SMD（Standardized Mean Difference）, ECOG（Eastern Cooperative Oncology Group）, GCB（germinal center B-cell）. Original queue: Pre-matching baseline characteristics of the LOW-DOX and HIGH-PLD groups. 1:1 matched queue: Post-matching characteristics after 1:1 PSM adjusting for covariates (age, LDH, Lugano stage, extranodal involvement, ECOG). Notes: Pre-matching significant difference in LDH (P<0.05); post-matching balance achieved (P>0.05). Post-matching SMD<0.1 for sex, LDH, extranodal involvement, Lugano stage, and ECOG; SMD<0.2 for age (>60 years).
