# Supplementary material for: Propensity-matched study of liposomal doxorubicin vs. doxorubicin in first-line DLBCL treatment: efficacy and safety
Source: Front Med (Lausanne). 2026 Apr 1;13:1769270. doi: 10.3389/fmed.2026.1769270 (PMC13079127; doi:10.3389/fmed.2026.1769270)
Supplement: Supplementary file 6 [file Table_6.docx]

| **Characteristic** | **LOW-DOX (n=47)** | **STD-DOX (n=323)** | **SMD** |
| --- | --- | --- | --- |
| Age, mean ± SD, years | 58.9 ± 15.2 | 51.4 ± 14.8 | 0.50 |
| Age ≥60 years, n (%) | 25 (53.2%) | 90 (27.9%) | 0.53 |
| Age ≥70 years, n (%) | 17 (36.2%) | 21 (6.5%) | 0.76 |
| Age ≥75 years, n (%) | 8 (17.0%) | — | — |
| ECOG PS ≥2, n (%) | 18 (38.3%) | 66 (20.4%) | 0.40 |
| LDH elevated, n (%) | 29 (61.7%) | 149 (46.1%) | 0.32 |
| Stage III–IV, n (%) | 29 (61.7%) | 176 (54.5%) | 0.15 |
| Extranodal involvement, n (%) | 18 (38.3%) | 59 (18.3%) | 0.45 |
| Male sex, n (%) | 26 (55.3%) | 171 (52.9%) | 0.05 |
| Hemoglobin <100 g/L, n (%) | 12/44 (27.3%) | — | — |
| ANC <1.5 ×10⁹/L, n (%) | 15/44 (34.1%) | — | — |
| Baseline LVEF, mean ± SD, % | 67.4 ± 3.5 | 68.0 ± 3.3 | 0.17 |
| LVEF <60%, n | 0 | — | — |
| DOX dose, mean ± SD, mg/m² | 32.4 ± 5.8 | 50.0 | — |
| Dose range, mg/m² | 20.8–39.7 | 50.0 | — |

**Table S7: Clinical Characteristics Associated with Doxorubicin Dose Reduction in the LOW-DOX Subgroup.**

Abbreviations: ANC, absolute neutrophil count; DOX, doxorubicin; ECOG PS, Eastern Cooperative Oncology Group performance status; LDH, lactate dehydrogenase; LVEF, left ventricular ejection fraction; SMD, standardized mean difference. Hemoglobin and ANC data available for 44 of 47 LOW-DOX patients.
