# Supplementary material for: Propensity-matched study of liposomal doxorubicin vs. doxorubicin in first-line DLBCL treatment: efficacy and safety
Source: Front Med (Lausanne). 2026 Apr 1;13:1769270. doi: 10.3389/fmed.2026.1769270 (PMC13079127; doi:10.3389/fmed.2026.1769270)
Supplement: Supplementary file 7 [file Table_7.docx]

|  |  |  | | Original queue | | | |  |  |  | 1:2 matching queue | | |  |  |
| --- | --- | --- | --- | --- | --- | --- | --- | --- | --- | --- | --- | --- | --- | --- | --- |
|  | LOW-DOX | | % | | LOW-PLD | % | P | SMD |  | LOW-DOX | % | LOW-PLD | % | P | SMD |
| N | 47 | |  | | 71 |  |  |  |  | 47 |  | 47 |  |  |  |
| Male sex | 26 | | (55.3) | | 45 | (63.4) | 0.494 | 0.165 |  | 26 | (55.3) | 29 | (61.7) | 0.675 | 0.13 |
| >60 years |  | |  | |  |  |  |  |  |  |  |  |  |  |  |
| No | 22 | | (46.8) | | 35 | (49.3) | 0.939 | 0.05 |  | 22 | (46.8) | 21 | (44.7) | 1 | 0.043 |
| Yes | 25 | | (53.2) | | 36 | (50.7) |  |  |  | 25 | (53.2) | 26 | (55.3) |  |  |
| Gene Expression Profiling |  | |  | |  |  |  |  |  |  |  |  |  |  |  |
| GCB | 26 | | (55.3) | | 33 | (46.5) | 0.657 | 0.129 |  | 26 | (60.5) | 20 | (51.3) | 0.539 | 0.186 |
| non-GCB | 17 | | (36.2) | | 28 | (39.4) |  |  |  | 17 | (39.5) | 19 | (48.7) |  |  |
| Unknown | 4 | | (8.5) | | 10 | (14.1) |  |  |  |  |  |  |  |  |  |
| Lactate dehydrogenase |  | |  | |  |  |  |  |  |  |  |  |  |  |  |
| Normal | 18 | | (38.3) | | 40 | (56.3) | 0.083 | 0.367 |  | 18 | (38.3) | 17 | (36.2) | 1 | 0.044 |
| Elevated | 29 | | (61.7) | | 31 | (43.7) |  |  |  | 29 | (61.7) | 30 | (63.8) |  |  |
| Lugano stage |  | |  | |  |  |  |  |  |  |  |  |  |  |  |
| I-II | 18 | | (38.3) | | 28 | (39.4) | 1 | 0.023 |  | 18 | (38.3) | 17 | (36.2) | 1 | 0.044 |
| III-IV | 29 | | (61.7) | | 43 | (60.6) |  |  |  | 29 | (61.7) | 30 | (63.8) |  |  |
| Number of extranodal sites |  | |  | |  |  |  |  |  |  |  |  |  |  |  |
| 0-1 | 29 | | (61.7) | | 50 | (70.4) | 0.432 | 0.185 |  | 29 | (61.7) | 29 | (61.7) | 1 | <0.001 |
| >2 | 18 | | (38.3) | | 21 | (29.6) |  |  |  | 18 | (38.3) | 18 | (38.3) |  |  |
| ECOG |  | |  | |  |  |  |  |  |  |  |  |  |  |  |
| 0-1 | 29 | | (61.7) | | 45 | (63.4) | 1 | 0.035 |  | 29 | (61.7) | 27 | (57.4) | 0.834 | 0.087 |
| 2-5 | 18 | | (38.3) | | 26 | (36.6) |  |  |  | 18 | (38.3) | 20 | (42.6) |  |  |

**Table S6．Baseline data of the LOW-DOX group and the LOW-PLD group before and after PSM 1:1 matching, n(%).** Abbreviations: LOW-DOX（low-dose DOX subgroup），LOW-PLD（low-dose PLD subgroup）, SMD（Standardized Mean Difference）, ECOG（Eastern Cooperative Oncology Group）, GCB（germinal center B-cell）. Original queue: Pre-matching baseline characteristics of the LOW-DOX and LOW-PLD groups. 1:1 matched queue: Post-matching characteristics after 1:1 PSM adjusting for covariates (age, LDH, Lugano stage, extranodal involvement, ECOG). Notes: No significant differences pre- or post-matching (P>0.05). Post-matching SMD<0.1 for all covariates (age, sex, LDH, Lugano stage, extranodal involvement, ECOG).
